# Osteoactivin (GPNMB) ectodomain protein promotes growth and invasive behavior of human lung cancer cells

**DOI:** 10.18632/oncotarget.7323

**Published:** 2016-02-11

**Authors:** Moses O. Oyewumi, Dharani Manickavasagam, Kimberly Novak, Daniel Wehrung, Nikola Paulic, Fouad M. Moussa, Gregory R. Sondag, Fayez F. Safadi

**Affiliations:** ^1^ Department of Pharmaceutical Sciences, College of Pharmacy, Northeast Ohio Medical University, Rootstown, OH 44272, USA; ^2^ School of Biomedical Sciences, Kent State University, Kent, OH 44240, USA; ^3^ Department of Anatomy and Neurobiology, College of Medicine, Northeast Ohio Medical University, Rootstown, OH 44272, USA

**Keywords:** GPNMB, cell adhesion, NSCLC, integrin, lung cancer

## Abstract

The potential application of GPNMB/OA as a therapeutic target for lung cancer will require a greater understanding of the impact of GPNMB/OA ectodomain (ECD) protein shedding into tumor tissues. Thus, in this work we characterized GPNMB/OA expression and extent of shedding of its ECD protein while evaluating the impact on lung cancer progression using three non-small cell lung cancer (NSCLC) cell lines: A549, SK-MES-1 and calu-6. We observed a direct correlation (*R*^2^ = 0.89) between GPNMB/OA expression on NSCLC cells and the extent of GPNMB/OA ECD protein shedding. Meanwhile, siRNA-mediated knockdown of GPNMB/OA in cancer cells significantly reduced GPNMB/OA ECD protein shedding, migration, invasion and adhesion to extracellular matrix materials. Also, exogenous treatment of cancer cells (expressing low GPNMB/OA) with recombinant GPNMB/OA protein (rOA) significantly facilitated cell invasion and migration, but the effects of rOA was negated by inclusion of a selective RGD peptide. Further studies in athymic (nu/nu) mice-bearing calu-6 showed that intratumoral supplementation with rOA effectively facilitated *in vivo* tumor growth as characterized by a high number of proliferating cells (Ki67 staining) coupled with a low number of apoptotic cells. Taken together, our results accentuate the relevance of GPNMB/OA ECD protein shedding to progression of lung cancer. Thus, strategies that suppress GPNMB/OA expression on lung cancer cells as well as negate shedding of GPNMB/OA ECD protein are worthy of consideration in lung cancer therapeutics.

## INTRODUCTION

Lung cancer is the most common cause of cancer death worldwide [1, 2]. In 2016, new cases in the United States could reach 230,000 with more than 150,000 deaths (cancer.org). Non-small cell lung cancer (NSCLC) accounts for 85% of all cases while small cell lung cancer (SCLC) accounts for about 15% of lung cancer cases. The prognosis is poor with less than 15% of patients surviving 5 years after diagnosis [3, 4]. The high mortality of lung cancer can be attributed to invasiveness and metastasis and the fact that the disease is not easily detectable until it reaches advanced stages [5]. Even though considerable improvements have been made in early diagnosis coupled with newly developed chemo/targeted therapies that improved responses, the overall 5-year survival for NSCLC cases remains low (15%) with a high rate of recurrence. It is widely accepted that tumor metastasis is a major barrier to treatment of lung cancer [6]. The prevalence of metastasis is demonstrated in several cases of lung cancer patients that have regional lymph-node involvement or distant disease at various stages of the disease [4]. In spite of the advances in treatment, cases of relapse and metastatic spread have created major hurdles to reaching the desired treatment outcome.

In this regard, treatment strategies that are based on epi (genetic) alterations of cancer cells (in isolation) have not delivered on the promise of improving survival of patients after diagnosis [7, 8]. Although, epi (genetic) alterations in tumor cells are essential (for tumor development), but are not sufficient to endow cancer cells with malignant properties [9]. It is now widely accepted that tumor cells acquire some of the essential features necessary for growth and metastatic dissemination through the ability to change their surroundings [9]. Thus, tumor cells create and maintain permissive environment for their development as a result of the cellular and non-cellular stroma components in tumor tissues [10]. In this study, we examined the expression and function of osteoactivin (OA)/(glycoprotein non-metastatic melanoma B; (GPNMB) in lung cancer progression.

GPNMB/OA is a type 1 transmembrane glycoprotein that is comprised of three domains: a long extracellular domain (ECD, ectodomain), a single transmembrane region and a short cytoplasmic domain [11–13]. GPNMB/OA has been identified as a melanosome-specific structural protein that shares high homology with Pme17, which is involved in many biological processes such as inflammation, tissue regeneration, cell migration and metastasis of malignant tumors [14]. Two GPNMB/OA mRNA isoforms encoding 560 and 572 amino acids have been identified that have a large ECD, a single pass transmembrane domain and a short cytoplasmic tail [15–17]. It has been reported that GPNMB/OA can be cleaved and shed from the cell surface, producing ECD fragments [18]. The ECD domain is made up of 12 potential N-glycosylation sites, a polycystic kidney disease (PKD) domain and a RGD motif [11, 15].

GPNMB/OA is expressed in a wide array of tissues and cell types including osteoblasts, osteoclasts in the bone as well as macrophages and dendritic cells in the hematopoietic system, melanocytes, keratinocytes, and Langerhans cells of the skin [19]. The biological role of GPNMB/OA has been linked to T-cells activation, fibroblast differentiation, and development of bone cells [13, 15]. Elevated expressions of GPNMB/OA have been shown to promote invasion and metastasis of prostate, hepatocellular carcinoma, glioma and breast cancer [12, 20–24]. In a study on glioblastoma multiforme patients, it was found that 35 out of 50 were positive (70%) for GPNMB/OA mRNA and 52 of 79 (66%) showed detectable GPNMB/OA in a membraneous and cytoplasmic pattern [16]. Another report showed that GPNMB/OA is highly and selectively expressed in aggressive bone-metastatic sub-populations of 4T1 breast cancer cells [24]. Although, considerable attention has been paid to characterize the function and clinical value of GPNMB/OA in various malignant tissues, however the expression and potential impact of GPNMB/OA ECD on the progression of lung cancer have not established. Concerning lung cancer, it was reported that GPNMB/OA overexpression was associated with decreased overall survival among small-cell lung cancer (SCLC) patients [25]. Specifically, it was reported that patients with weak GPNMB/OA expression had an average survival time of 27 months as compared to 15 months among patients with high GPNMB/OA expression [25]. Meanwhile, potential application of GPNMB/OA as a therapeutic target for lung cancer will require an understanding on the implication of GPNMB/OA ECD protein shedding in lung cancer growth and progression. In this regard, we hypothesized that overexpression of GPNMB/OA on lung cancer cells will directly result in increased shedding of GPNMB/OA ECD protein that will facilitate lung cancer growth. It has been reported that GPNMB/OA protein acts as a growth factor in inducing MMP-3 expression via ERK pathway in fibroblasts [21, 26]. Also supporting the premise of this work is the report that the GPNMB/OA ECD showed potential angiogenic properties in breast cancer [18]. This earlier work also provided insights into a possible mechanism by which the ECD domain of GPNMB/OA is shed. The investigators showed that GPNMB/OA ECD protein is cleaved and shed (primarily mediated by ADAM-10) from the tumor cell surface, producing ECD fragments [18]. In general, members of ADAM are up-regulated in most cancers and are responsible for shedding many substrates that have been implicated in cancers [27–29]. Thus, in this work that the pro-tumor and pro-metastatic function of GPNMB/OA in lung cancer is most likely propagated through shedding of the ECD protein into tumor tissues which could play a major role in creating favorable environment that supports tumor growth.

In this regard, we characterized GPNMB/OA expression levels and shedding of its ECD protein in selected NSCLC cell lines while assessing the effects of GPNMB/OA ECD protein on cell migration, invasion and adhesion to extracellular matrix materials (ECM). In order to mimic the process of GPNMB/OA ECD protein shedding into tumor tissues, we developed an *in-vivo* tumor model in athymic (nu/nu) mice with or without exogenous supplementation of recombinant GPNMB/OA (rOA) that represents the ECD protein [11, 30, 31]. The information generated from the work may be relevant in assessing the pro-tumor and pro-metastasis functions of GPNMB/OA ECD protein that is shed into tumor tissues according to GPNMB/OA expression levels.

## RESULTS

### Characterization of GPNMB/OA expression in lung cancer cells

**Figure 1:**
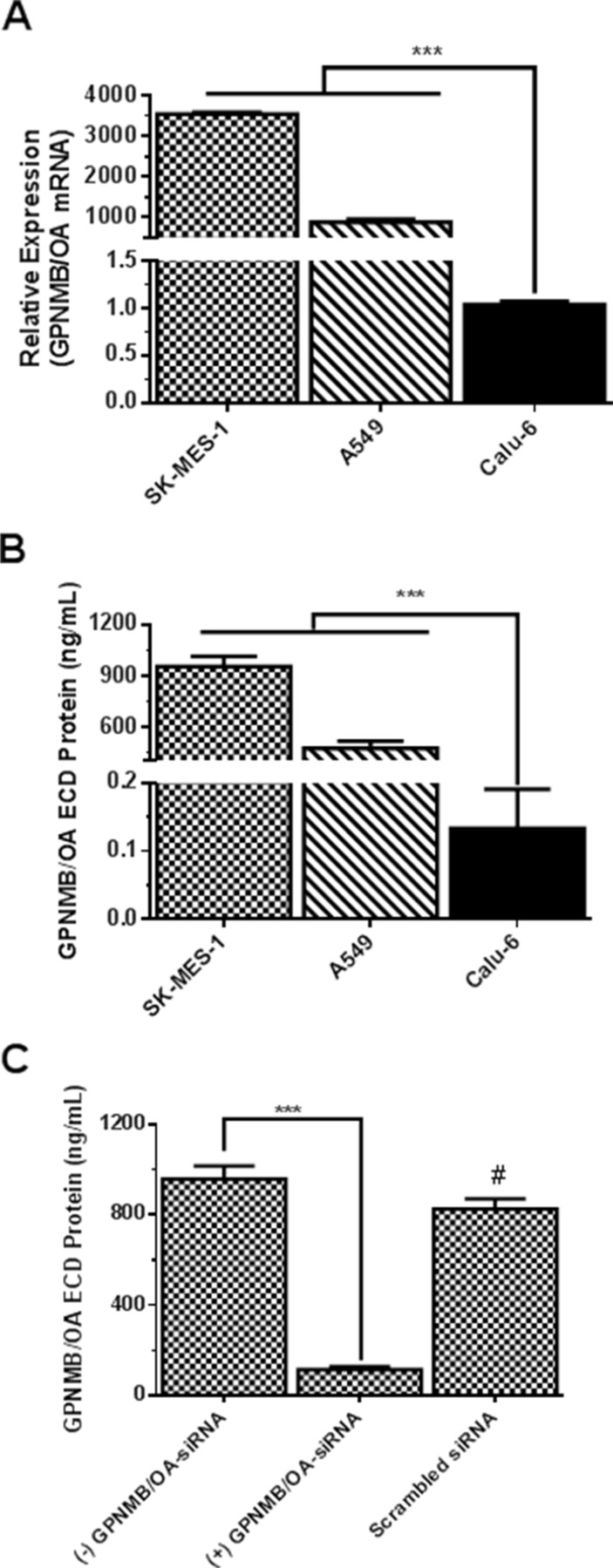
Characterization of GPNMB/OA expression in lung cancer cell lines (**A**) GPNMB/OA mRNA levels (mean ± SD; *n* = 4) in lung cancer cell lines as determined by qPCR (***p < 0.001 for SK-MES-1 and A549 cells versus calu-6). (**B**) The extent of GPNMB/OA ECD protein shedding (24 hr) into the conditioned media by SK-MES-1, A549 and calu-6 cells as determined by ELISA (mean ± SD; *n* = 6). The extent of GPNMB/OA ECD protein shedding in SK-MES-1 and A549 cells was significantly higher than calu-6 cells (***p < 0.001). (**C**) Knockdown of GPNMB/OA expression (mean ± SD; *n* = 4) resulted in a significant reduction (***p < 0.001) in GPNMB/OA ECD in conditioned media of SK-MES-1. #*p* > 0.05 when comparing scrambled siRNA-transfected cells versus control cells (−) GPNMB/OA-siRNA.

The expression levels of GPNMB/OA in three representative NSCLC cell lines were determined. These cell lines are: SK-MES-1 (squamous carcinoma cell line) and A549 cells (human adenocarcinoma cell line) that are known to be metastatic in comparison to an anaplastic carcinoma cell line (calu-6 cells) (that are known be weakly metastatic). The levels of GPNMB/OA mRNA in SK-MES-1, A549 and calu-6 cells are shown in Figure 1A. Both SK-MES-1 and A549 cells showed significantly higher GPNMB/OA mRNA levels compared to calu-6 cells (Figure 1A). We observed that the GPNMB/OA mRNA levels in the cells correlated very well with the extent of GPNMB/OA ECD protein that was shed into the conditioned media of each cell line. As measured by ELISA, SK-MES-1 cells showed the highest level of GPNMB/OA ECD protein shedding into the conditioned media (Figure 1B). Meanwhile, calu-6 cells had a negligible level of GPNMB/OA ECD protein shedding compared to SK-MES-1 and A549 cells (Figure 1B). Further data analysis showed a strong linear correlation (*R*^2^ = 0.89) between GPNMB/OA mRNA level and the level of GPNMB/OA ECD protein shed into the conditioned media (Figure 1A and 1B, Supplementary Figure S1). For instance, calu-6 cells that showed the least level of GPNMB/OA mRNA also produced little or no GPNMB/OA ECD protein shedding. We later examined if the level of GPNMB/OA ECD protein levels in SK-MES-1 will diminish with siRNA-mediated knockdown of GPNMB/OA expression. As shown in Figure 1C, we observed that suppression of GPNMB/OA expression in SK-MES-1 cells resulted in diminished levels of GPNMB/OA ECD protein shedding into the conditioned media (*p* < 0.001, Figure 1C). Further, SK-MES-1 cells that were transfected with control siRNA (scrambled siRNA) did not have a marked effect on ECD protein shedding (*p* > 0.05; Figure 1C). The results demonstrated that shedding of GPNMB/OA ECD protein is dictated by GPNMB/OA mRNA expression level in the representative NSCLC cells.

### GPNMB/OA promotes invasive and metastatic behavior in lung cancer cells

**Figure 2:**
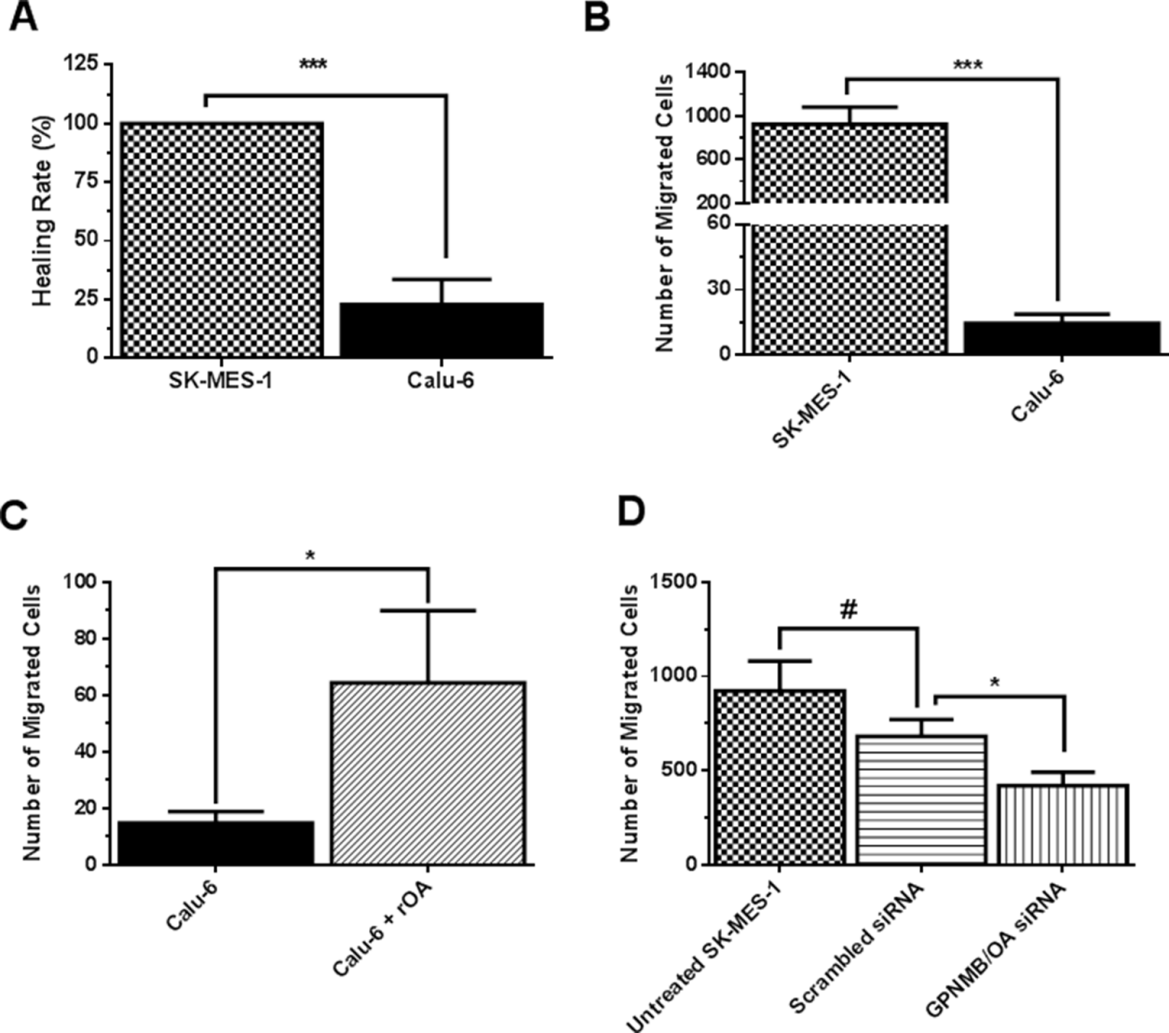
Effects of GPNMB/OA on lung cancer cell invasion and migration (**A**) The percent healing rates (mean ± SD; *n* = 5–6) for SK-MES-1 and calu-6 cells at 24 hr after inflicting wound into the cell monolayers (***p < 0.001 versus calu-6 cells). (**B**) The number of migrated cells (24 hr, mean ± SD; *n* = 5–6) for SK-MES-1 and calu-6 cell lines using Transwell migration assay. (***p < 0.001 for SK-MES-1 cells versus calu-6 cells). (**C**) The number of migrated cells (24 hr, mean ± SD; *n* = 5–6) in transwell insert for calu-6 cells that were seeded with or without rOA (50 ng/mL). Calu-6 cells that were seeded with rOA showed a higher number of migrated cells versus control calu-6 cells (*p < 0.05). (**D**) The number of migrated cells (mean ± SD; *n* = 3–4) in transwell for SK-MES-1 with siRNA-mediated knockdown of GPNMB/OA was significantly reduced (*p < 0.05) compared to control cells with endogenous GPNMB/OA expression.

We conducted a set of experiments to investigate whether GPNMB/OA over-expression will support invasive and aggressive behaviors in lung cancer cells. To accomplish this goal, we selected SK-MES-1 as a high GPNMB/OA expressing cell line while calu-6 was a low GPNMB/OA expressing cell line. Observations from *in-vitro* scratch assay showed that calu-6 cells were less effective (compared to SK-MES-1 cells) in migrating to fill up the wound area as indicated from the healing rate (Figure 2A). The percentage healing rate for calu-6 cells (that produced the least amount of GPNMB/OA ECD protein) was 4.5 times lower than SK-MES-1 cells (Figure 2A). A similar trend was observed from transwell migration assay in that a higher number of SK-MES-1 cells migrated compared to calu-6 cells (*p* < 0.001; Figure 2B). In order to assess the impact of GPNMB/OA ECD protein, we conducted cell migration and invasion studies in the presence of exogenous supplementation of rOA (a prototype of GPNMB/OA ECD [9, 28, 29]). Calu-6 cells that were seeded with or without rOA supplementation (50–100 ng/mL), we conducted transwell migration assay. The average number of migrated cells after rOA supplementation was about 4 times higher than cells that did not receive rOA (*p* < 0.05, Figure 2C). In order to confirm the link between cell migration and GPNMB/OA expression, we conducted transwell migration studies using SK-MES-1 cells with siRNA-mediated suppression of GPNMB/OA expression levels (Figure 2D). While cells that were transfected with scrambled siRNA did not show detectable changes in cell migration, we observed that SK-MES-1 cells that were transfected with GPNMB/OA siRNA showed a marked reduction in cell migration (*p* < 0.05; Figure 2D). The data indicated that GPNMB/OA expression level could be linked to the extent to which cell invasion and migration are facilitated.

### *In-vitro* assessment of functional implication of exogenous GPNMB/OA protein treatment

**Figure 3:**
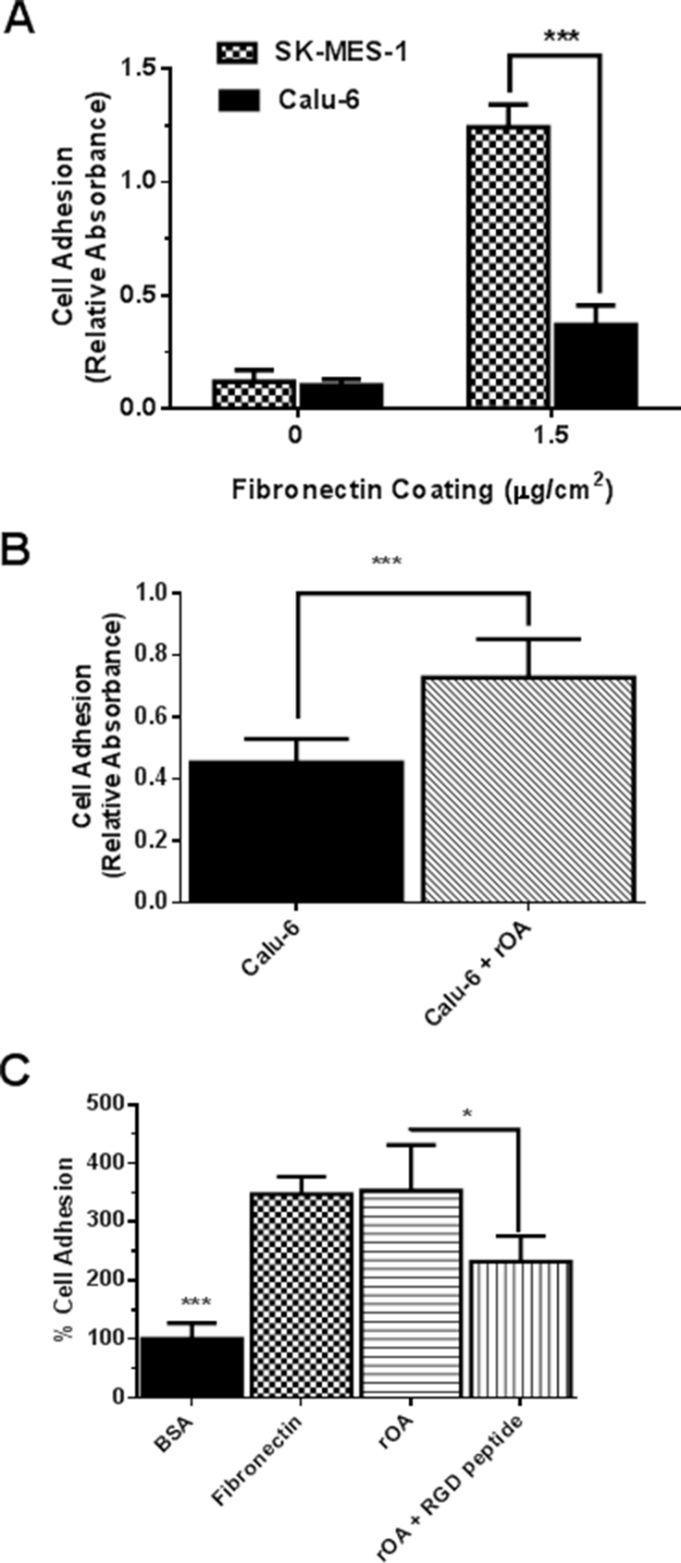
GPNMB/OA ECD protein promoted cell adhesion to fibronectin-coated or rOA-coated plates (**A**) Adhesion of SK-MES-1 and calu-6 cells (mean ± SD; *n* = 4–5) to fibronectin-coated plates at 37°C (***p < 0.001 for SK-MES-1 versus calu-6 cells). The extent of cell adhesion expressed as absorbance values was quantified using MTT dye. (**B**) Adhesion (mean ± SD; *n* = 6) of calu-6 cells to fibronectin-coated plates with or without rOA protein (50 ng/mL) treatment. (***p < 0.001; for calu-6 cells that were seeded with rOA versus control calu-6 cells. The extent of cell adhesion expressed as absorbance values was quantified using MTT dye. (**C**) Characterization (mean ± SD; *n* = 6) of calu-6 cell adhesion to culture plates coated with 1% BSA (negative control), fibronectin (8 μg/mL, positive control) and rOA (8 μg/mL, with or without RGD selective peptide). Cell adhesion to BSA-coated plates was significantly less than both fibronectin and rOA-coated plates. Cell treatment with a selective RGD peptide resulted in a significant reduction in cell adhesion to rOA-coated plates (*p < 0.05). The extent of cell adhesion expressed as fluorescence intensity was quantified by CyQuant assay.

Tumor cell adhesion to extracellular matrices and basement membranes is considered to be a crucial step in the invasive process for metastatic cells. Thus, we examined the influence of rOA on the adhesion of calu-6 (a low GPNMB/OA-expressing cell line) and SK-MES-1 (a high GPNMB/OA-expressing cell line) cells to the substrates pre-coated with fibronectin, which is a major basement membrane component. Without fibronectin coating, both calu-6 and SK-MES-1 cells showed negligible attachment to cell culture plates (Figure 3A). As expected for both SK-MES-1 and calu-6, the extent of cell adhesion increased significantly with fibronectin coating. A significantly higher number of SK-MES-1 cells attached to fibronectin-coated plates than calu-6 cells (*p* < 0.001, Figure 3A). For calu-6 cells, rOA supplementation resulted in a significant increase in cell adhesion to fibronectin-coated plates (*p* < 0.001; Figure 3B). Additional studies showed that cell adhesion to rOA-coated plates was comparable to fibronectin-coated plates at levels that were significantly higher than BSA-coated plates (Figure 3C). We also observed that co-treatment of cells with selective RGD peptide markedly diminished the extent of cell adhesion to rOA-coated plates (Figure 3C).

In a follow-up study, culture plates that were coated with varying concentrations of rOA were used in cell adhesion studies (Supplementary Figure S2). There was a marked increase in cell adhesion as the concentration of rOA used in coating culture plates increased from 1 to 2 μg/mL, but the effect on cell adhesion tampered off as rOA concentrations increased from 2 to 8 μg/mL (Supplementary Figure S2).

### Intratumoral treatments with GPNMB/OA ECD protein facilitated *in-vivo* tumor growth

**Figure 4:**
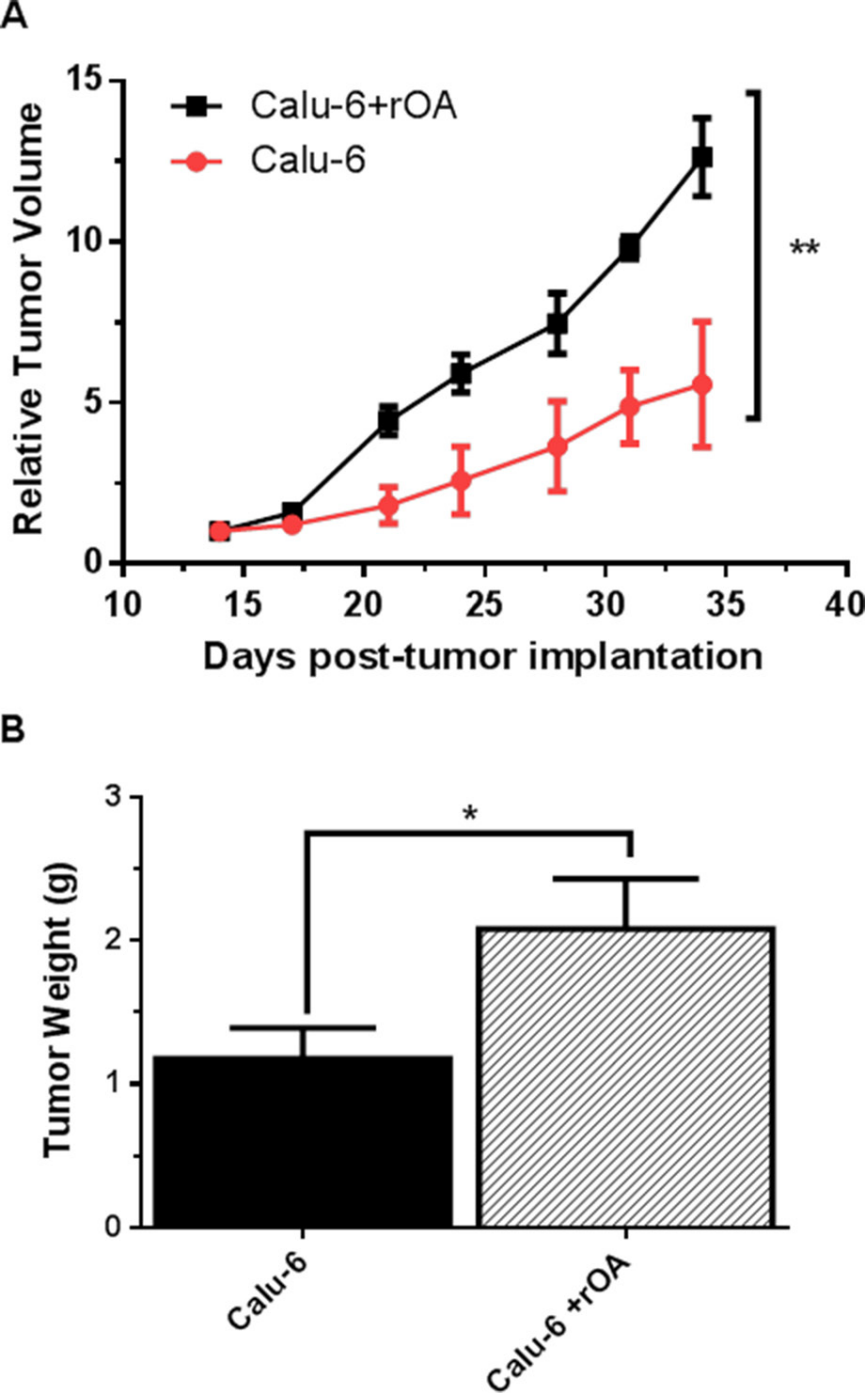
GPNMB/OA protein promoted *in-vivo* lung cancer growth (**A**) Calu-6 tumor growth in athymic (nu/nu) mice as expressed by relative tumor volume (mean ± SD; *n* = 4–5 mice). Relative tumor volumes for calu-6 tumors with rOA were significantly larger than calu-6 cells developed with PBS (calu-6; **p < 0.01). (**B**) Tumor weights (mean ± SD; *n* = 4–5 mice) measured on day 34 post-tumor implantation in athymic (nu/nu) mice. Calu-6 tumors developed with rOA supplementation resulted in significantly bigger tumors (*p < 0.05) compared to calu-6 tumors without rOA.

**Figure 5:**
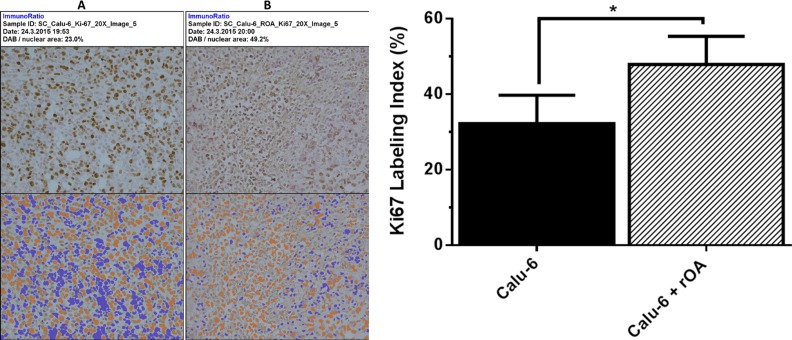
Immunohistochemical examination of cell proliferation in tumor tissues (**A**–**B**) Representative tumor sections stained for cell proliferation based on for ki67 expression on day 34 post-tumor implantation in athymic *nu/nu* mice. The extent of ki67 staining was processed by ImmunoRatio for control calu-6 tumors (without exogenous rOA, with PBS) (A); and calu-6 tumors that received exogenous rOA supplementation (B). Top panel: original image. Bottom panel: original image with segmented staining components for quantification of Ki67 staining. (**C**) Quantification of Ki67 staining of tumor tissues based on percentage of positively stained nuclear area as processed with ImmunoRatio. *(*p* < 0.05, mean ± SD; *n* = 8 images) for calu-6 cells that were treated with exogenous rOA versus control calu-6 tumors.

**Figure 6:**
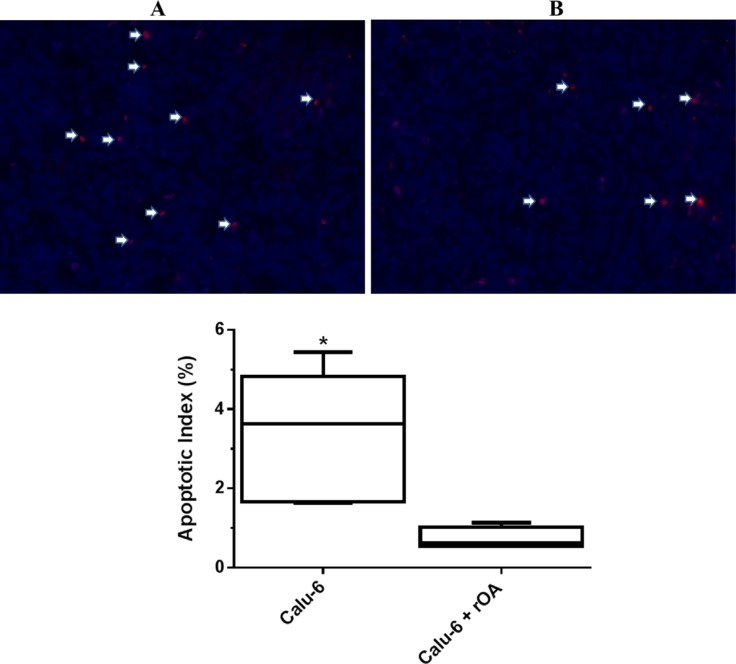
Extent of apoptosis in calu-6 tumor tissues that received exogenous rOA supplementation (**A**) Representative tumor sections stained for apoptosis using TUNEL assay on day 34 post-tumor implantation in athymic *nu/nu* mice. Arrows indicate TUNEL positive cells for control calu-6 tumors (PBS-treated) (A) and calu-6 tumors that received exogenous rOA supplementation (**B**). (**C**) Quantification of TUNEL staining of tumor tissues excised on day 34 from calu-6 tumor-bearing athymic (nu/nu) mice. Data-points are plotted as the percentage of apoptotic (TUNEL positive) cells (mean ± SD; *n* = 6 images). *(*p* < 0.05) for calu-6 cells that were treated with exogenous rOA versus control calu-6 tumors.

Evaluation of the effect of GPNMB/OA ECD protein on *in-vivo* tumor growth was conducted in athymic (nu/nu) mice bearing calu-6 tumor. It was considered that since calu-6 cells produced the least amount of GPNMB/OA ECD protein, we expect that intratumoral injections of rOA could mimic the situation where GPNMB/OA ECD protein is shed into tumor tissues. In this regard, tumor-bearing athymic mice were distributed into two groups: (i) calu-6 tumor alone with intratumoral injection of PBS; and (ii) calu-6 tumors with rOA supplementation. The progression of tumor growth was monitored up till day 34 after tumor implantation. We observed that intratumoral rOA supplementation resulted in a significant (*p* < 0.01, Figure 4A) increase in tumor volumes compared to calu-6 tumors developed with PBS. A similar trend was observed for resultant tumor weights in that rOA supplementation resulted in tumors with bigger weights compared to calu-6 tumors with PBS supplementation (*p* < 0.05; Figure 4B). In each group, animal weights were maintained during the course of tumor growth (Supplementary Figure S3). To better characterize the impact of rOA supplementation on tumor growth, the excised tumor tissues were assessed by H & E and immunohistochemical (IHC) examinations. A high power magnification view of H & E sections from calu-6 tumors with or without rOA supplementation showed that tumor sections from both animal groups showed variations in size and shape of nuclei with occasional mitosis (Supplementary Figure S4–S5). Sections from tumors developed with rOA supplementation showed apparent highly dense sheets of cells while sections from tumors without rOA supplementation alone appeared less dense with high numbers of vacuoles (Supplementary Figure S5). A clear distinction between the two animal groups was observable from the IHC staining of excised tumors. Further, we applied Ki-67 staining to confirm the extent of proliferation in the tumor sections (Figure 5). Tumors that received rOA supplementation showed a significantly higher Ki-67 labeling index compared to control tumors without rOA (*p* < 0.05; Figure 5A–5C). Similarly, TUNEL staining was used to detect the number of apoptotic cells in each tumor section (Figure 6). Calu-6 tumors without rOA supplementation showed a higher number of apoptotic cells compared to control tumors (Figure 6A–6C). In all, quantification of the immunohistochemical staining showed that calu-6 tumors developed with rOA supplementation reflected a lower amount of apoptotic and a higher number of proliferative tumor cell (Figures 5 and 6).

## DISCUSSION

The current work is based on the premise that potential consideration of GPNMB/OA as a therapeutic target for lung cancer will require a deeper understanding of potential impact of GPNMB/OA expression and shedding of its ECD protein. We paid particular attention to NSCLC cell lines (SK-MES-1, A549 and calu-6 cells) since NSCLC contributes about 80–85% of all lung cancer cases. Our observation highlights the relevance of GPNMB/OA ECD protein shedding in lung cancer progression. Specifically, there was a direct correlation between GPNMB/OA mRNA level and GPNMB/OA ECD protein shedding. The trend connotes that lung cancer cells with high GPNMB/OA mRNA expression level should be expected to secrete more GPNMB/OA ECD protein.

We also investigated whether the effect of GPNMB/OA in promoting invasive and aggressive behavior in tumors will be propagated via secretion of GPNMB/OA ECD protein. This is particularly important since earlier studies on other types of cancers (hepatocellular carcinoma, glioma and breast cancer) have shown that GPNMB/OA is expressed at higher levels in several malignant human tissues relative to corresponding normal tissues [12, 16, 20, 24]. In this regard, we conducted *in-vitro* scratch, invasion and migration assays. Our data indicated that GPNMB/OA ECD protein facilitated cell invasion, migration and healing rate of wound inflicted into cell monolayers. For instance, SK-MES-1 cells, with high levels of GPNMB/OA ECD protein shedding, showed correspondingly a higher invasive and aggressive behavior compared to calu-6 cells (with low levels of GPNMB/OA ECD protein shedding). It is noteworthy that the number of migrated calu-6 cells increased significantly with exogenous supplementation of rOA (a prototype of GPNMB/OA ECD [9, 28, 29]). Further studies showed that silencing GPNMB/OA expression in lung cancer cells markedly diminished the extent of GPNMB/OA ECD shedding and invasive behavior (the number of migrated cells). Thus, it is likely that the function of GPNMB/OA in promoting invasive and aggressive behavior in lung cancer cells was achieved through shedding of its ECD protein.

Further characterization of the link between GPNMB/OA and invasive behavior of lung cancer was assessed by cell adhesion studies. Particularly, we evaluated the extent to which lung cancer cell adhesion to a model ECM (fibronectin) will increase in the presence or absence of exogenous rOA supplementation. It is reported that an initial step in the invasive process for metastatic tumor cells involves an increased adhesion to ECM [32–34]. Using fibronectin as a model of ECM, cells that secrete more GPNMB/OA ECD protein adhere more than cells that secrete low GPNMB/OA ECD protein. Meanwhile, adhesion of cells that do not produce GPNMB/OA ECD protein was significantly increased with exogenous supplementation with rOA (a porotype of GPNMB/OA ECD protein. Thus, it is possible that enhancement cell adhesion to ECM by GPNMB/OA ECD protein contributed to the aggressive behavior of lung cancer cells.

To assess the role of GPNMB/OA ECD in promoting *in-vivo* tumor growth, we developed tumor xenografts in athymic (nu/nu) mice using calu-6 cells (with low GPNMB/OA mRNA expression). Calu-6 cells were selected for tumor xenograft studies because the cells produce low levels of GPNMB/OA ECD protein; such that the impact of exogenous rOA intratumor supplementation can be observable. Compared to systemic administration of rOA, we considered that intratumoral rOA injections would mimic the shedding of GPNMB/OA ECD protein into tumor tissues. We were able to deduce that intratumor injection was effective in retaining rOA within the tumor tissues for local effect since we did not find detectable human GPNMB/OA protein levels (ELISA) in mouse blood samples obtained from mice bearing calu-6 alone or calu-6 with rOA supplementation (data not shown). Also, rOA supplementation did not induce elevation of mouse serum GPNMB/OA protein levels because the serum levels of mouse GPNMB/OA protein were at comparable levels in all animal groups (Supplementary Figure S8). Our results indicated that GPNMB/OA ECD was effective in propagating the growth of calu-6 tumor xenograft in athymic (nu/nu) mice. Data from histomorphological and immunohistochemical analyses of excised tumor tissues showed that calu-6 tumors that received rOA intratumoral supplementation showed lower apoptotic index and higher proliferation (ki67 staining) compared to tumors that did not receive rOA supplementation. The data demonstrated that exogenous supplementation with GPNMB/OA ECD protein was most probably effective in modifying the tumor microenvironment that supported tumor growth and aggressive behavior.

The involvement of GPNMB/OA ECD protein in promoting enhancement of cell migration and adhesion may be relevant to the pro-tumor and/or pro-metastatic functions of GPNMB/OA. It is reported that when cancer cells become metastatic, they develop altered affinity and avidity for the extracellular matrix [32–34]. Since GPNMB/OA ECD protein has a RGD binding domain [11], it is likely that GPNMB/OA ECD protein promotes cell adhesion to ECM via possible interaction with RGD binding integrins. The impact of RGD motif on GPNMB/OA ECD protein is further supported by our observation that inclusion of a selective RGD peptide significantly reduced the extent of cell adhesion to rOA-coated plates. Additional mechanistic studies are warranted to elucidate the effects of GPNMB/OA ECD protein on lung cancer growth as well as the impact of possible interaction of GPNMB/OA ECD protein with RGD-binding integrins on lung cancer progression. Our observation is supported by previous reports that the integrin-binding RGD domain is required for the GPNMB/OA-dependent adhesive interaction between melanocytes and keratinocytes [35]. The possibility that GPNMB/OA ECD protein-integrin interaction contributed to lung cancer growth was also demonstrated in a recent report that GPNMB/OA cooperates with neutropilin-1 to promote mammary tumor growth and engages integrin α5β1 for efficient breast cancer metastasis [32]. Another plausible explanation is that GPNMB/OA ECD may modulate MMPs activity based on an earlier work that showed stimulation of NIH-3T3 fibroblasts with a recombinant GPNMB/OA ECD displayed an enhanced Erk and p38 phosphorylation along with upregulation of Mmp-3 mRNA [21]. The findings from our work thus suggest that potential application of GPNMB/OA as a therapeutic target for lung cancer should incorporate strategies that will inhibit the mechanism by which GPNMB/OA ECD protein is shed or negate the effects of the GPNMB/OA ECD protein in tumor tissues.

In summary, we have characterized the expression and function of GPNMB/OA in three NSCLC cell lines (SK-MES-1, A549 and calu-6 cells) with the following key observations: (i) the shedding of GPNMB/OA ECD protein by NSCLC cells is directly correlated with GPNMB/OA mRNA levels; (ii) GPNMB/OA ECD protein plays an influential role in promoting cell migration, invasion and adhesion to a model ECM; and (iii) shedding of GPNMB/OA ECD protein to tumor tissues may contribute directly to the progression of lung cancer based on studies in athymic (nu/nu) mice. Additional mechanistic studies are warranted to fully elucidate the role of GPNMB/OA ECD protein in lung cancer progression. The work reported herein underscores the importance of suppressing GPNMB/OA expression and/or negating GPNMB/OA ECD protein shedding as potential therapeutic strategies in lung cancer.

## MATERIALS AND METHODS

### Materials

Eagle's minimum essential medium (EMEM), Transwell inserts, phosphate buffered saline (PBS), and dimethyl sulfoxide (DMSO), CyQuant assay kit were obtained from Fisher Scientific (Pittsburg, PA). Cell culture plates (6, 24, and 96 wells) were obtained from USA Scientific (Ocala, FL). The following were obtained from Sigma Aldrich (St. Louis, MO): Bovine serum albumin (BSA) Dulbecco's modified eagle medium (DMEM), thiazolyl blue tetrazolium bromide (MTT), 4′,6-diamidino-2-phenylindole dihydrochloride (DAPI), bovine serum albumin, and penicillin/streptomycin. Fetal bovine serum (FBS) was acquired from Atlanta Biologicals (Lawrenceville, GA). Dharmafect 1 transfection reagent, OA siRNA (ON-TARGETplus SMARTpool), and non-targeting (scrambled) siRNA (ON-TARGETplus non-targeting control siRNA) were obtained from G.E. Dharmacon (Lafayette, CO). Recombinant GPNMB/OA (rOA), ELISA kits (human OA, murine OA) were obtained from R & D Systems (Minneapolis, MN). A selective RGD (Arg-Gly-Asp) blocking peptide was purchased from Peptides International, Louisville, KY.

### Cell culture

Human lung cancer cell lines A549, SK-MES-1, and calu-6 were obtained from American Type Culture Collection (ATCC, Manassas, VA), and maintained in a humidified incubator (37°C, 5% CO_2_). A549 cells were maintained in Dulbecco's modified eagle medium (DMEM) supplemented with 10% FBS and antibiotics (100 μg/mL streptomycin and 100 units/mL penicillin). Similarly, SK-MES-1 and calu-6 cells were cultured in Eagle's minimum essential medium (EMEM) containing 10% FBS and antibiotics (100 μg/mL streptomycin and 100 units/mL penicillin).

### Cell adhesion assay

Cell adhesion assay was performed with modification of the procedure previously reported [30, 36]. Briefly, 96 well plates were coated with fibronectin (BD Bioscience, San Jose, CA) using the manufacture's procedure. To coat the plates, 50 μL of fibronectin solution (30 μg/mL) was added to each well and incubated at room temperature for 1 hr. Residual fibronectin solution was aspirated, and the wells were rinsed with distilled water. Non-specific binding sites were blocked by incubating with BSA (0.5% w/v) for 30 min at room temperature. After rinsing with PBS, cells were seeded (5 × 10^4^ cells/well) in serum free media with or without rOA (50–100 ng/mL). To allow the cells to attach, the plate was incubated at 37°C for 40 min. Non-adherent cells were removed by carefully aspirating the supernatant and rinsing with PBS. Complete growth media containing MTT dye was then introduced and incubated for 4 hr (37°C). The supernatant was aspirated, and the reduced formazan dye was solubilized by the addition of DMSO. The absorbance was immediately measured on a microplate reader (Spectramax-340PC, Bucher Biotec AG, Basel, Switzerland), and the data points were expressed as the mean optical density at 570 nm. For assessment of cell attachment to rOA-coated plates, 96-well plates were coated with varying concentrations of rOA (0–8 μg/mL). After saturating non-specific binding sites with BSA (0.5% w/v) for 30 min at room temperature, the cells were seeded (5 × 10^4^ cells/well) in serum free media. Non-adherent cells were removed by carefully aspirating the supernatant and rinsing with PBS. To quantify the number of cells adhered to rOA-coated plates; we applied CyQuant assay kit (Molecular Probes) according to the manufacturer's protocol.

### Scratch (wound healing) assay

The wound healing assay was conducted using an established procedure [37]. Cells were seeded in a 24 well plate (1.75 × 10^5^ cells/well) and allowed to adhere overnight before the monolayer was wounded by scratching with a 200 μL pipette tip. To assess the effects of rOA, cells were seeded with rOA (100 ng/mL) and allowed to adhere overnight before inflicting wound in the monolayer. After wounding the cell monolayer, detached cells were removed by rinsing with PBS before media containing 1% FBS was added to each well. Images of the scratch were acquired immediately after wounding (0 hr) and 24 hr later. The healing rate was calculated using the closure ratio analysis method [38]: healing rate (%) = [(0 hr scratch area minus 24 hr scratch area)/0 hr scratch area] × 100.

### *In-vitro* invasion and migration assays

Cellular invasion and migration were assessed using Transwell inserts (8 μm pore diameter) as reported previously with minor modification [24]. Cells were seeded in serum free media (5 × 10^5^ cells/well) alone or with rOA (100 ng/mL) in the upper chambers of inserts that had been pre-coated with BD Matrigel^™^ (BD Bioscience, San Jose, CA). The receiving chamber was filled with growth media containing 10% FBS. After incubating at 37°C for 48 hr, the upper chamber was swabbed with a Q-tip, and cells that had invaded through the membrane were fixed with paraformaldehyde and stained with DAPI. The number of invasive cells was then counted using an Axio Zoom V16 microscope (Zeiss, Thornwood, NY). Migration was assessed using the same procedure with the following modifications: (i) the seeding density was 2.5 × 10^4^ cells/well, (ii) the cells were allowed to migrate for 24 hr, and (iii) the Matrigel coating was omitted.

### Knockdown of GPNMB/OA expression

Transient suppression of GPNMB/OA expression was achieved by transfecting A549 and SK-MES-1 cells with OA-targeted siRNA (ON-TARGETplus SMARTpool siRNA). Dharmafect 1 transfection reagent and OA-targeted siRNA (50 nM) were incubated with cells for 24 hr (37°C) before being aspirated and replaced with serum free media. After incubating in serum free media for 24 hr at 37°C the conditioned media was collected, and the cells lysed with RIPA buffer (Boston Bioproducts, Ashland, MA) containing protease inhibitor cocktail-2 (Sigma Aldrich, St. Louis, MO). Control cells were transfected with non-targeting (scrambled) siRNA (ON-TARGETplus non-targeting control siRNA).

### Western blotting

Using standard western blotting procedure, cells were lysed in RIPA buffer containing protease inhibitors. Samples were centrifuged at 14,000 rpm for 45 min at 4^°^C. Protein concentration from the supernatant was measured by Pierce BCA Protein Assay Kit (Thermo Scientific, Rockford, IL). One dimensional SDS-polyacrylamide gel electrophoresis was performed with a corresponding gel concentration using discontinuous buffer system of Laemmli (Bio-Rad Laboratories, Richmond, CA). The electrophoresed proteins were transferred to a polyvinylidene difluoride membrane and subjected to immunoblot analysis with antibody to acetylated tubulin (Santa Cruz Biotechnology). The reaction was detected with enhanced chemiluminescence (Amersham Life Science, Arlington Heights, IL). The blot was reprobed with tubulin antibody after washing to check for equal loading of the gel.

### Quantitative PCR analysis

RNA was isolated as described [39]. Cell monolayers were harvested and homogenized in Trizol. The homogenized cell monolayers were separated by chloroform while RNA was recovered using isopropyl alcohol precipitation. Pellets were washed with 70% ethanol and RNA concentrations were determined. For qPCR, 2 μg of total RNA was reverse transcribed to cDNA. Two microliters of cDNA was amplified in 50 μL of qPCR reaction mixture. The primers for human GPNMB/OA were used Forward: 5′-CTGTGAACACAGCCAATGTG-3′ and Reverse: 5′-ATGGGGAGATCTTTGAGGAA-3′. qPCR reactions were performed in ABI PRISM 7700 using SYBR Green method. GPNMB/OA values were calculated relative to G3PDH values.

### Animal studies

In accordance with NIH guidelines, the animal use protocol was approved by the NEOMED Animal Care and Use Committee. Tumors were developed in female athymic *nu/nu* mice (6–8 weeks old; Charles River Laboratories, Wilmington, MA) by subcutaneous administration of calu-6 cells as we have previously reported [40]. Briefly, mice were randomly assigned to one of two treatment groups: (i) calu-6 cells alone with intratumoral injections (50 uL injection volume) of PBS (calu-6), and (ii) calu-6 cells with intratumoral injections of rGPNMB/OA (rOA) supplementation (calu-6 + rOA). To develop the tumor, calu-6 cells (3 × 10^6^ cells/mouse; suspended in BD Matrigel^™^) were injected into the interscapular region of each mouse (day 0). In the group with rOA supplementation, we administered by intratumoral injection, rOA protein (50 ng rOA/injection) on days 0, 26, 33, 40, and 47. Each animal's body weight and tumor volume were measured twice each week throughout the course of the study. Tumor volume (V_tumor_) was determined by measuring the diameter of each tumor in two perpendicular axes, denoted as the length (L) and width (W) using Vernier calipers, and calculated as V_tumor_ = ½ (L·W^2^). The relative tumor volume (V_Rel_) for each mouse was defined as the ratio of the tumor volume two weeks after deposition of calu-6 cells (V_T(initial)_) to the tumor volume at later time points (V_T(x)_) using the equation V_Rel_ = V_T(x)_/V_T(initial)_. When the tumor volumes had exceeded 2,000 mm^3^, the mice were sacrificed, and the tissues and whole blood were collected. Excised tissues were rinsed in PBS before being fixed in 4% paraformaldehyde at 4°C, and subsequently embedding in paraffin as previously described [40]. The weight of the tumors and lungs was recorded prior to fixation. Whole blood, collected by cardiac puncture, was centrifugation at 1,500 × g for 15 min (4°C) to obtain plasma for GPPNMB/OA measurement by ELISA (R & D Systems, Minneapolis, MN).

### Histological and immunohistochemical assessments of tumor tissues

Paraffin embedded tissues were sectioned (5 μm), deparaffinized, and rehydrated before hematoxylin and eosin (H & E) staining. Tumor sections were also subjected to additional analysis via immunohistochemistry (IHC) labeling of Ki-67, GPNMB/OA and terminal dUTP nick end-labeling (TUNEL). The IHC was performed with the following antibodies: Ki-67 (1:100 dilution; clone MIB-1, DAKO M7240), and GPNMB/OA (1:100; Bioss Inc; bs2684R). TUNEL staining was performed using the *In-Situ* Cell Death Detection Kit (Roche Applied Science, Indianapolis, IN) in accordance with the manufacturer's instructions. Sections labeled with TUNEL were counterstained with DAPI before imaging. The percentage of apoptotic cells was determined by dividing the number of TUNEL positive cells by the total number of cells [41]. Likewise, quantitative analysis of Ki-67 staining was carried out using immunoRatio, which is a calculation the percentage of positively stained nuclear area (labeling index) by using a color deconvolution algorithm [42, 43].

### Data analysis

Statistical analysis was carried out using Student's *t*-test or a single factor ANOVA. Student-Newman-Keuls post-hoc test was applied for multiple comparisons while assessing significance at *p*-value of < 0.05. The graphs were prepared with GraphPad Prism 6 (GraphPad Software, CA).

## SUPPLEMENTARY MATERIALS FIGURES



## References

[1] Oyewumi MO, Alazizi A, Wehrung D, Manochakian R, Safadi FF (2014). Emerging lung cancer therapeutic targets based on the pathogenesis of bone metastases. Int J Cell Biol.

[2] Parkin DM, Bray F, Ferlay J, Pisani P (2005). Global cancer statistics, 2002. CA Cancer J Clin.

[3] Bulk E, Sargin B, Krug U, Hascher A, Jun Y, Knop M, Kerkhoff C, Gerke V, Liersch R, Mesters RM, Hotfilder M, Marra A, Koschmieder S (2009). S100A2 induces metastasis in non-small cell lung cancer. Clin Cancer Res.

[4] Hirsch FR, Franklin WA, Gazdar AF, Bunn PA (2001). Early detection of lung cancer: clinical perspectives of recent advances in biology and radiology. Clin Cancer Res.

[5] Miller YE (2005). Pathogenesis of lung cancer: 100 year report. Am J Respir Cell Mol Biol.

[6] Miller RE, Jones JC, Tometsko M, Blake ML, Dougall WC (2014). RANKL inhibition blocks osteolytic lesions and reduces skeletal tumor burden in models of non-small-cell lung cancer bone metastases. J Thorac Oncol.

[7] Dai SP, Xie C, Ding N, Zhang YJ, Han L, Han YW (2015). Targeted inhibition of genome-wide DNA methylation analysis in epigenetically modulated phenotypes in lung cancer. Med Oncol.

[8] Zhou C, Qin Y, Xie Z, Zhang J, Yang M, Li S, Chen R (2015). NPTX1 is a novel epigenetic regulation gene and associated with prognosis in lung cancer. Biochem Biophys Res Commun.

[9] Hanahan D, Weinberg RA (2011). Hallmarks of cancer: the next generation. Cell.

[10] Scherz-Shouval R, Santagata S, Mendillo ML, Sholl LM, Ben-Aharon I, Beck AH, Dias-Santagata D, Koeva M, Stemmer SM, Whitesell L, Lindquist S (2014). The reprogramming of tumor stroma by HSF1 is a potent enabler of malignancy. Cell.

[11] Maric G, Rose AA, Annis MG, Siegel PM (2013). Glycoprotein non-metastatic b (GPNMB): A metastatic mediator and emerging therapeutic target in cancer. Onco Targets Ther.

[12] Rose AA, Grosset AA, Dong Z, Russo C, Macdonald PA, Bertos NR, St-Pierre Y, Simantov R, Hallett M, Park M, Gaboury L, Siegel PM (2010). Glycoprotein nonmetastatic B is an independent prognostic indicator of recurrence and a novel therapeutic target in breast cancer. Clin Cancer Res.

[13] Safadi FF, Xu J, Smock SL, Rico MC, Owen TA, Popoff SN (2001). Cloning and characterization of osteoactivin, a novel cDNA expressed in osteoblasts. J Cell Biochem.

[14] Zhou LT, Liu FY, Li Y, Peng YM, Liu YH, Li J (2012). Gpnmb/osteoactivin, an attractive target in cancer immunotherapy. Neoplasma.

[15] Abdelmagid SM, Barbe MF, Rico MC, Salihoglu S, Arango-Hisijara I, Selim AH, Anderson MG, Owen TA, Popoff SN, Safadi FF (2008). Osteoactivin, an anabolic factor that regulates osteoblast differentiation and function. Exp Cell Res.

[16] Kuan CT, Wakiya K, Dowell JM, Herndon JE, Reardon DA, Graner MW, Riggins GJ, Wikstrand CJ, Bigner DD (2006). Glycoprotein nonmetastatic melanoma protein B, a potential molecular therapeutic target in patients with glioblastoma multiforme. Clin Cancer Res.

[17] Moss ML, Stoeck A, Yan W, Dempsey PJ (2008). ADAM10 as a target for anti-cancer therapy. Curr Pharm Biotechnol.

[18] Rose AA, Annis MG, Dong Z, Pepin F, Hallett M, Park M, Siegel PM (2010). ADAM10 releases a soluble form of the GPNMB/Osteoactivin extracellular domain with angiogenic properties. PloS one.

[19] Vaklavas C, LoBuglio A, Saleh M, Yelin M, Forero A, Phillips GL (2013). CDX-011 (Glembatumumab Vedotin, CR011-vcMMAE). Antibody-Drug Conjugates and Immunotoxins.

[20] Fiorentini C, Bodei S, Bedussi F, Fragni M, Bonini SA, Simeone C, Zani D, Berruti A, Missale C, Memo M, Spano P, Sigala S (2014). GPNMB/OA protein increases the invasiveness of human metastatic prostate cancer cell lines DU145 and PC3 through MMP-2 and MMP-9 activity. Exp Cell Res.

[21] Furochi H, Tamura S, Mameoka M, Yamada C, Ogawa T, Hirasaka K, Okumura Y, Imagawa T, Oguri S, Ishidoh K, Kishi K, Higashiyama S, Nikawa T (2007). Osteoactivin fragments produced by ectodomain shedding induce MMP-3 expression via ERK pathway in mouse NIH-3T3 fibroblasts. FEBS Lett.

[22] Onaga M, Ido A, Hasuike S, Uto H, Moriuchi A, Nagata K, Hori T, Hayash K, Tsubouchi H (2003). Osteoactivin expressed during cirrhosis development in rats fed a choline-deficient, L-amino acid-defined diet, accelerates motility of hepatoma cells. J Hepatol.

[23] Qian X, Mills E, Torgov M, LaRochelle WJ, Jeffers M (2008). Pharmacologically enhanced expression of GPNMB increases the sensitivity of melanoma cells to the CR011-vcMMAE antibody-drug conjugate. Mol Oncol.

[24] Rose AA, Pepin F, Russo C, Abou Khalil JE, Hallett M, Siegel PM (2007). Osteoactivin promotes breast cancer metastasis to bone. Mol Cancer Res.

[25] Li YN, Zhang L, Li XL, Cui DJ, Zheng HD, Yang SY, Yang WL (2014). Glycoprotein nonmetastatic B as a prognostic indicator in small cell lung cancer. APMIS.

[26] Ogawa T, Nikawa T, Furochi H, Kosyoji M, Hirasaka K, Suzue N, Sairyo K, Nakano S, Yamaoka T, Itakura M, Kishi K, Yasui N (2005). Osteoactivin upregulates expression of MMP-3 and MMP-9 in fibroblasts infiltrated into denervated skeletal muscle in mice. Am J Physiol Cell Physiol.

[27] Parkin E, Harris B (2009). A disintegrin and metalloproteinase (ADAM)-mediated ectodomain shedding of ADAM10. J Neurochem.

[28] Arribas J, Bech-Serra JJ, Santiago-Josefat B (2006). ADAMs, cell migration and cancer. Cancer Metastasis Rev.

[29] Duffy MJ, Mullooly M, O'Donovan N, Sukor S, Crown J, Pierce A, McGowan PM (2011). The ADAMs family of proteases: new biomarkers and therapeutic targets for cancer?. Clin Proteomics.

[30] Moussa FM, Hisijara IA, Sondag GR, Scott EM, Frara N, Abdelmagid SM, Safadi FF (2014). Osteoactivin promotes osteoblast adhesion through HSPG and alphavbeta1 integrin. J Cell Biochem.

[31] Sondag GR, Salihoglu S, Lababidi SL, Crowder DC, Moussa FM, Abdelmagid SM, Safadi FF (2014). Osteoactivin induces transdifferentiation of C2C12 myoblasts into osteoblasts. J Cell Physiol.

[32] Desgrosellier JS, Cheresh DA (2010). Integrins in cancer: biological implications and therapeutic opportunities. Nat Rev Cancer.

[33] Pal S, Ganguly KK, Chatterjee A (2013). Extracellular matrix protein fibronectin induces matrix metalloproteinases in human prostate adenocarcinoma cells PC-3. Cell Commun Adhes.

[34] Pal S, Moulik S, Dutta A, Chatterjee A (2014). Extracellular matrix protein laminin induces matrix metalloproteinase-9 in human breast cancer cell line mcf-7. Cancer microenvironment.

[35] Yoon HJ, Cho YR, Joo JH, Seo DW (2013). Knockdown of integrin alpha3beta1 expression induces proliferation and migration of non-small cell lung cancer cells. Oncol Rep.

[36] Sun Y, Lu N, Ling Y, Gao Y, Chen Y, Wang L, Hu R, Qi Q, Liu W, Yang Y, You Q, Guo Q (2009). Oroxylin A suppresses invasion through down-regulating the expression of matrix metalloproteinase-2/9 in MDA-MB-435 human breast cancer cells. Eur J Pharmacol.

[37] Liang CC, Park AY, Guan JL (2007). *In vitro* scratch assay: a convenient and inexpensive method for analysis of cell migration *in vitro*. Nat Protoc.

[38] Chen Y, Peng W, Lu Y, Chen J, Zhu YY, Xi T (2013). MiR-200a enhances the migrations of A549 and SK-MES-1 cells by regulating the expression of TSPAN1. J Biosci.

[39] Abdelmagid SM, Barbe MF, Arango-Hisijara I, Owen TA, Popoff SN, Safadi FF (2007). Osteoactivin acts as downstream mediator of BMP-2 effects on osteoblast function. J Cell Physiol.

[40] Wehrung D, Bi L, Geldenhuys WJ, Oyewumi MO (2013). Antitumor efficacy and tolerability of systemically administered gallium acetylacetonate-loaded gelucire-stabilized nanoparticles. J Biomed Nanotechnol.

[41] Pei XM, Yung BY, Yip SP, Ying M, Benzie IF, Siu PM (2014). Desacyl ghrelin prevents doxorubicin-induced myocardial fibrosis and apoptosis via the GHSR-independent pathway. Am J Physiol Endocrinol Metab.

[42] Remes SM, Tuominen VJ, Helin H, Isola J, Arola J (2012). Grading of neuroendocrine tumors with Ki-67 requires high-quality assessment practices. The Am J Surg Pathol.

[43] Tuominen VJ, Ruotoistenmaki S, Viitanen A, Jumppanen M, Isola J (2010). ImmunoRatio: a publicly available web application for quantitative image analysis of estrogen receptor (ER), progesterone receptor (PR), and Ki-67. Breast Cancer Res.

