# Supplementary material for: Osteoactivin (GPNMB) ectodomain protein promotes growth and invasive behavior of human lung cancer cells
Source: Oncotarget. 2016 Feb 11;7(12):13932–44. doi: 10.18632/oncotarget.7323 (PMC4924689; doi:10.18632/oncotarget.7323)
Supplement: Supplementary file 1 [file oncotarget-07-13932-s001.pdf]

Osteoactivin (GPNMB) ectodomain protein promotes growth and invasive behavior of human lung cancer cells

Supplementary Materials

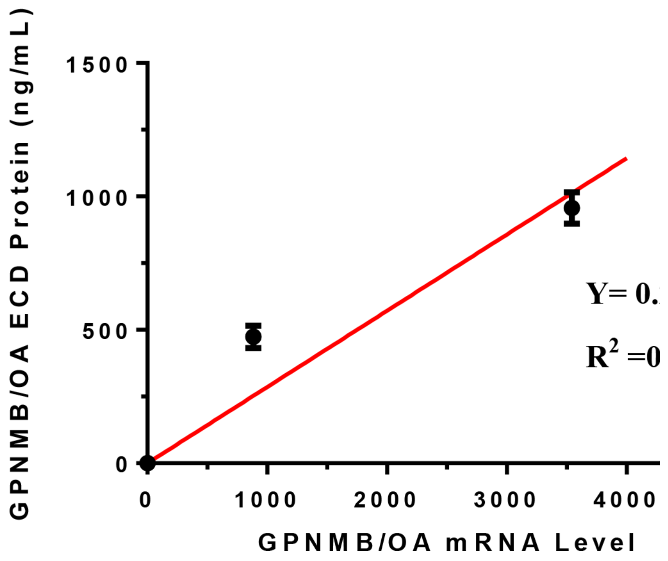

Supplementary Figure S1: Linear correlation between GPNMB/OA mRNA levels and extent of GPNMB/OA ECD protein that was shed (24 hr) into the conditioned media of SK-MES-1, A549 and calu-6 cells.

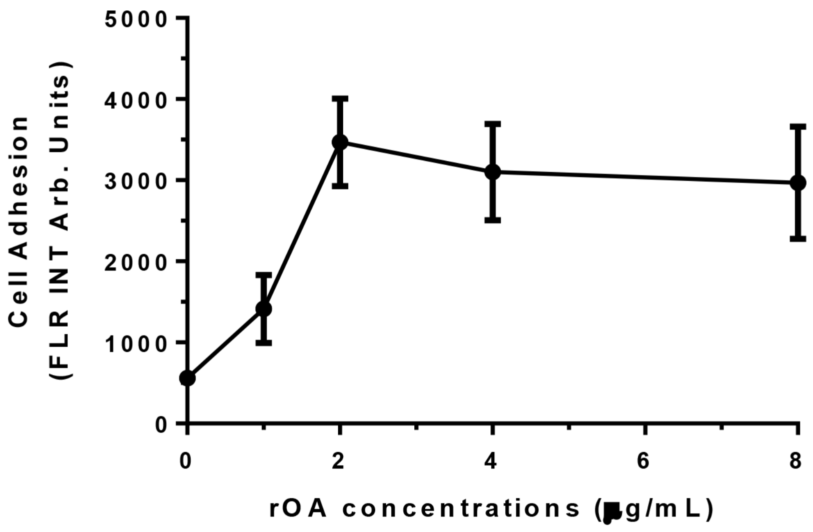

Supplementary Figure S2: Adhesion (mean ± SD; *n* = 6) of calu-6 cells to rOA-coated plates (1–8 µg/mL). Cell adhesion to rOA-coated plates (2–8 µg/mL) was significantly higher than uncoated plates (***$p < 0.001$). The extent of cell adhesion was measured by Alamar blue assay.

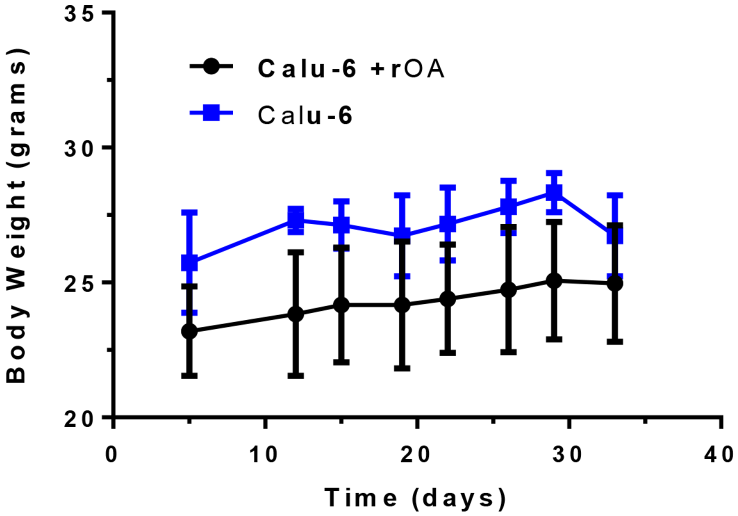

Supplementary Figure S3: Animal body weights as measured during the course of *in-vivo* tumor progression. Calu-6 tumor xenografts were developed in athymic (nu/nu) mice. The animals were divided into groups that received intra-tumor injections of PBS (calu-6) and those that received intra-tumor injection of rOA (calu-6 + rOA). Each data-point represents the mean ± SD (n = 4–5 mice).

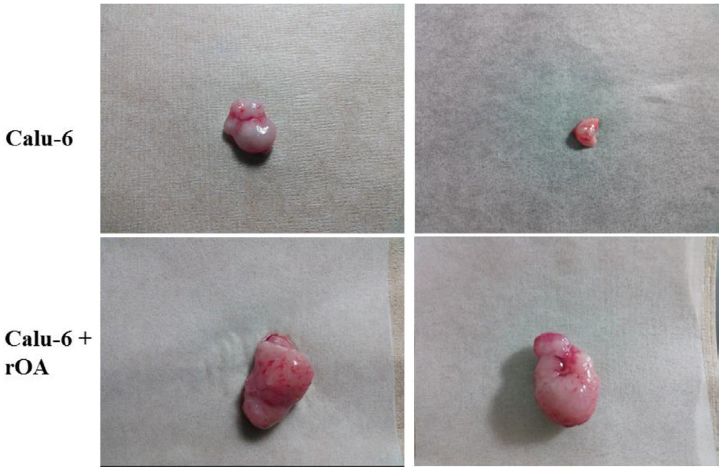

Supplementary Figure S4: Representative images of excised tumors obtained from athymic (nu/nu) mice-bearing calu-6 tumors that received intratumor treatments of PBS (calu-6) or rOA (calu-6 + rOA). The animals were sacrificed on day 34 post tumor implantation.

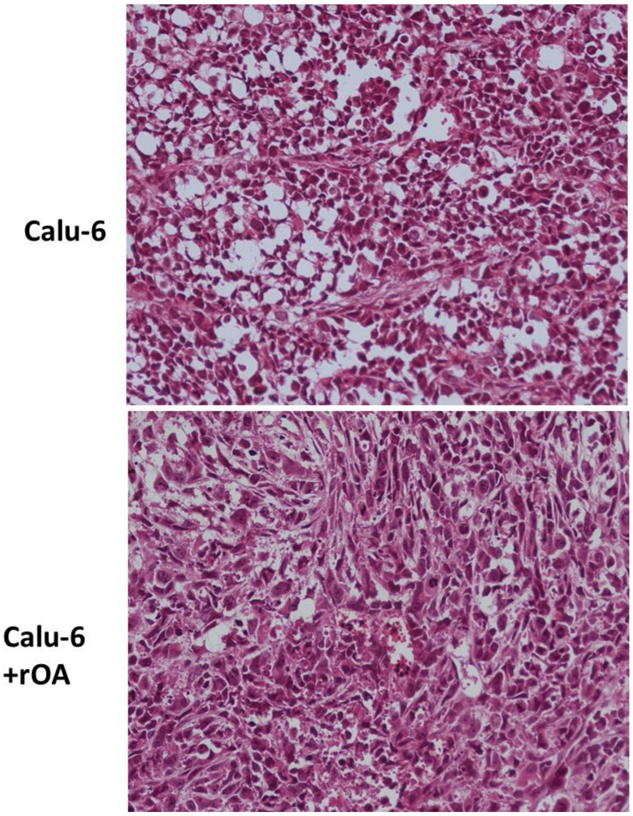

Supplementary Figure S5: Representative H/E images of tumor sections obtained from athymic (nu/nu) mice-bearing calu-6 tumors that received intratumor treatments of PBS (calu-6) or rOA. The animals were sacrificed on day 34 post tumor implantation.

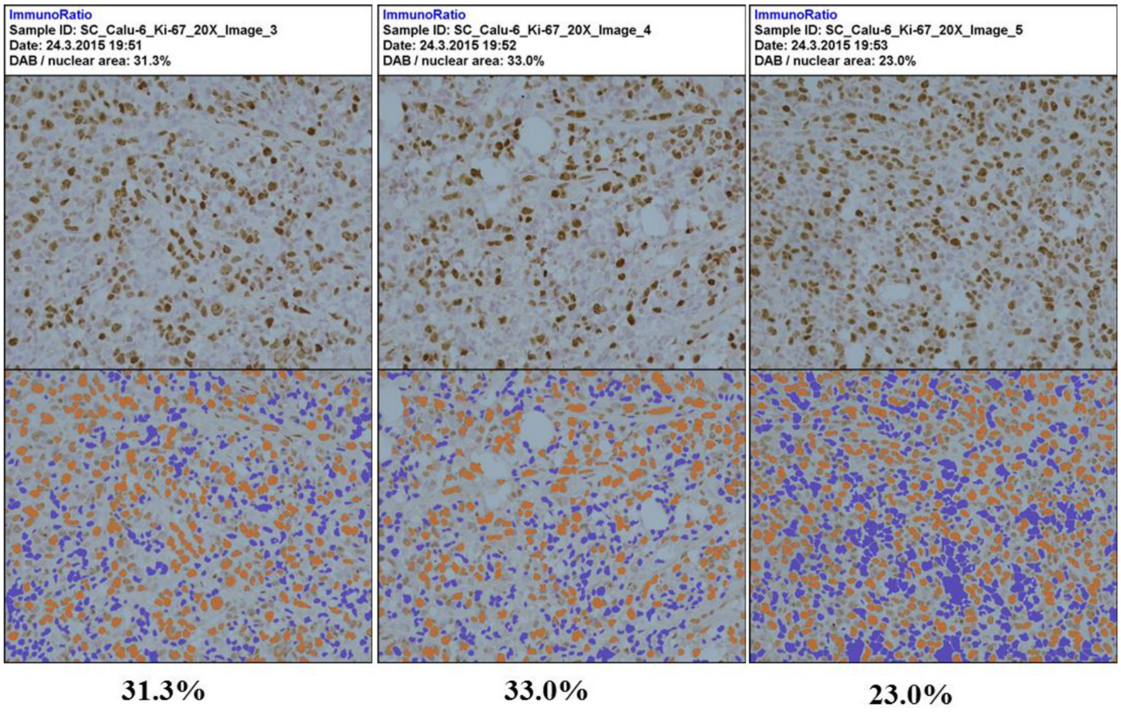

Supplementary Figure S6: The extent of Ki-67 staining for three representative tumor sections obtained from athymic (nu/nu) mice-bearing calu-6 tumor that received intratumor injections of PBS. The extent of ki-67 staining based on labeling index was processed by ImmunoRatio and found to be 31.3%, 33.0% and 23.0% as indicated. Top panel: original image. Bottom panel: original image with segmented staining components for quantification of Ki67 staining.

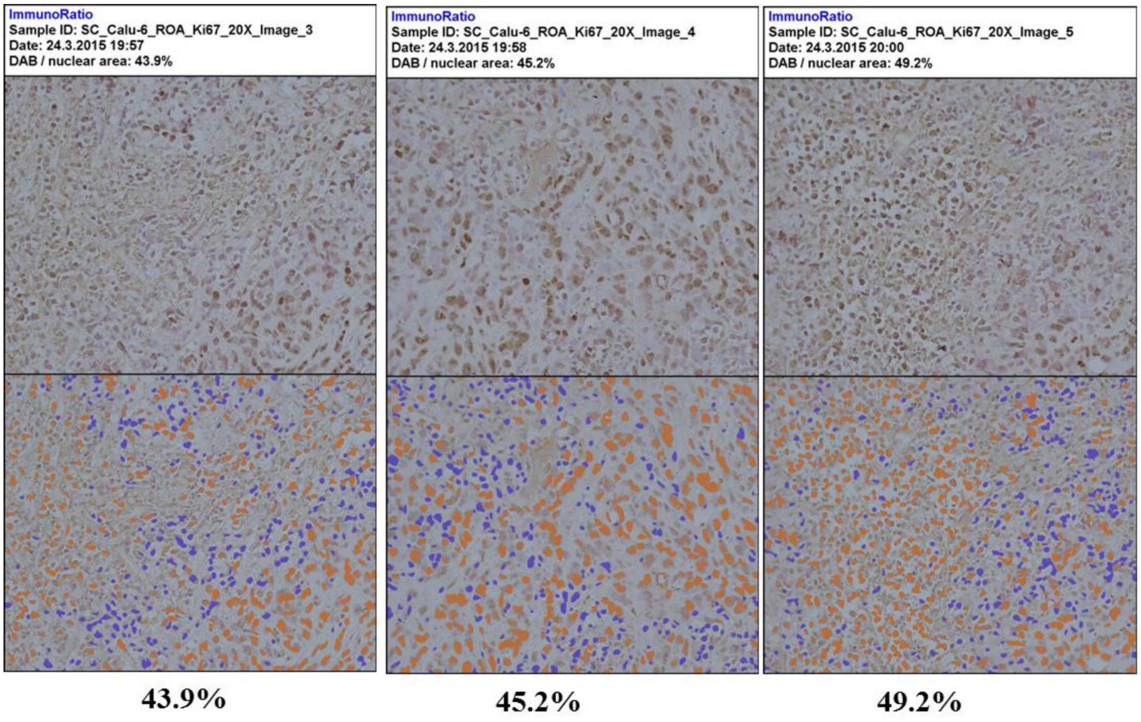

Supplementary Figure S7: The extent of Ki-67 staining for three representative tumor sections obtained from athymic (nu/nu) mice-bearing calu-6 tumor that received intratumor injections of rOA. The extent of ki-67 staining based on labeling index was processed by ImmunoRatio and found to be 31.3%, 33.0% and 23.0% as indicated. Top panel: original image. Bottom panel: original image with segmented staining components for quantification of Ki67 staining.

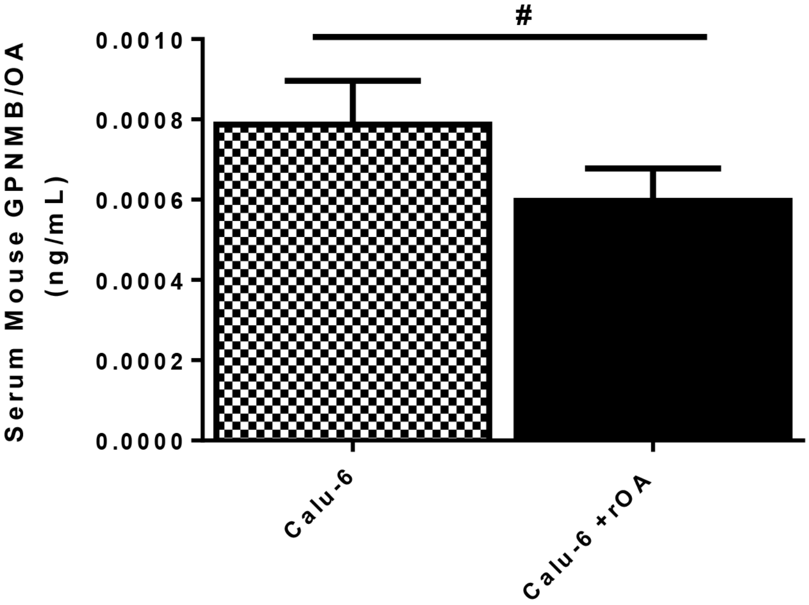

Supplementary Figure S8: The serum levels of mouse GPNMB/OA in athymic (nu/nu) mice-bearing calu-6 tumors as measured by ELISA. In each mouse, the serum sample was obtained on day 34 after tumor implantation. #represent $p > 0.05$ for calu-6 tumors with or without rOA supplementation. Each data-point represents the mean ± SD; n = 3–4 mice.
